# Long noncoding RNA NEAT1 promotes laryngeal squamous cell cancer through regulating miR-107/CDK6 pathway

**DOI:** 10.1186/s13046-016-0297-z

**Published:** 2016-01-29

**Authors:** Peng Wang, Tianyi Wu, Han Zhou, Qianqian Jin, Guoqing He, Haoyang Yu, Lijia Xuan, Xin Wang, Linli Tian, Yanan Sun, Ming Liu, Lingmei Qu

**Affiliations:** Department of Otorhinolaryngology, Head and Neck Surgery, The Second Affiliated Hospital, Harbin Medical University, No. 246, Baojian Road, Harbin, 150086 China; Department of Otorhinolaryngology, Head and Neck Surgery, The Fifth Affiliated Hospital, Harbin Medical University, No. 213, Jianshe Road, Daqing, China

**Keywords:** Laryngeal squamous cell cancer, Long noncoding RNA, microRNA, NEAT1, CDK6, miR-107

## Abstract

**Background:**

Long noncoding RNA nuclear paraspeckle assembly transcript 1 (NEAT1) plays key role in the progression of some human cancers. However, the role of NEAT1 in human laryngeal squamous cell cancer (LSCC) is still unknown. We therefore investigated the expression and function of NEAT1 in LSCC.

**Methods:**

NEAT1 level in LSCC and adjacent non-neoplastic tissues were detected by qRT-PCR. NEAT1 was knockdown in LSCC cells and cell proliferation, apoptosis and cell cycle were examined. The growth of xenografts with NEAT1 knockdown LSCC cells was analyzed.

**Results:**

NEAT1 level was significantly higher in LSCC than in corresponding adjacent non-neoplastic tissues, and patients with neck nodal metastasis or advanced clinical stage had higher NEAT1 expression. Moreover, siRNA mediated NEAT1 knockdown significantly inhibited the proliferation and induced apoptosis and cell cycle arrest at G1 phase in LSCC cells. The growth of LSCC xenografts was significantly suppressed by the injection of NEAT1 siRNA lentivirus. Furthermore, NEAT1 regulated CDK6 expression in LSCC cells which was mediated by miR-107.

**Conclusion:**

NEAT1 plays an oncogenic role in the tumorigenesis of LSCC and may serve as a potential target for therapeutic intervention.

## Background

Laryngeal squamous cell carcinoma (LSCC) is one of the most common malignancies in the head and neck [1]. Despite the improvements in diagnostic and therapeutic modalities, the survival of laryngeal cancer patients is still poor over the past 20 years. Therefore, revealing the molecular mechanism underlying LSCC development is important for developing effective therapy. Recently, we and others have focused on the role of aberrant expression of noncoding RNAs in LSCC [2–6]. Noncoding RNAs, which do not encode protein, include short noncoding RNAs and long noncoding RNAs (lncRNAs). By binding to specific sequences usually in the 3′ untranslated region (3′UTR) to degrade the transcription of target genes, microRNAs have been demonstrated to play important role in multiple cancers including LSCC [2–7]. Long noncoding RNAs are a class of transcripts longer than 200 nucleotides. Recent studies have shown that lncRNAs are frequently dysregulated in cancer progression [8, 9]. Nuclear paraspeckle assembly transcript 1 (NEAT1) lncRNA is found to localize specifically to paraspeckles which has two isoforms: 3.7 kb NEAT1-1 and 23 kb NEAT1-2 [10]. They share the same 5′-end but are processed alternatively at the 3′-termini. NEAT1 may serve as a core structural component because knockdown of NEAT1 led to the disintegration of paraspeckles [10]. NEAT1 upregulation has been reported in human malignancies, including leukemia, hepatocellular carcinoma and lung cancers [11–13]. However, little is known about whether NEAT1 affects carcinogenesis and biological behavior of LSCC. Our preliminary experiments with microarray analysis showed that NEAT1 was upregulated in LSCC tissues (data not shown). In the present study, we further detected the expression of NEAT1 in LSCC by using real-time PCR and found that lncRNA NEAT1 was up-regulated and correlated with the progression of LSCC. Next, we investigated the function of NEAT1 in LSCC cells and found that NEAT1 knockdown inhibited the proliferation and invasion of LSCC cells. Furthermore, we demonstrated that NEAT1 upregulated cyclin-dependent kinase 6 (CDK6) through inhibiting the expression of miR-107. These results provide the evidence that NEAT1-miR-107-CDK6 regulatory network promotes LSCC.

## Methods

### Tissue samples

Pairs of tumor tissues and adjacent non-cancerous matched tissues were obtained from 52 patients who underwent partial or total laryngectomy between August 2013 and December 2014 at the Department of Otorhinolaryngology, the Second Affiliated Hospital of Harbin Medical University, under an approved protocol of Harbin Medical University. The patients had not received any cancer therapy before admission. After surgery, the tissues obtained from patients were preserved in liquid nitrogen within 5 min of excision and then were transported frozen to the laboratory and stored at −80 °C.

### Quantitative real-time PCR analysis

Total RNA was extracted by using Trizol reagent (Invitrogen, Carlsbad, CA, USA). 2 μg of total RNA were reversely transcribed to cDNA by using High Capacity cDNA Reverse Transcription Kits (Applied Biosystems, Foster City, CA, USA). Quantitative PCR was performed using Power SYBR Green RT-PCR Reagents (Applied Biosystems, Foster City, CA, USA). Primers were as follows: NEAT1 forward, 5′-CTTCCTCCCTTTAACTTATCCATTCAC-3′; reverse, 5′-CTCTTCCTCCACCATTACCAACAATAC-3′; miR-107 forward, 5′-ATGATGAGCAGCATTGTACAGG-3′; reverse, 5′-GCAGGGTCCGAGGTATTC-3′. All reactions were carried out on the Applied Biosystems 7000 Sequence Detection System (Applied Biosystems, Foster City, CA, USA). Relative mRNA and lncRNA levels were normalized against β-actin and U6 snRNA, respectively, and calculated using the 2^-ΔΔCt^ method.

### Cell culture and virus transduction

Human LSCC cell line Hep-2 was cultured in Dulbecco’s modified Eagle’s medium containing 10 % fetal bovine serum (Gibco; Life Technologies, Carlsbad, CA) in a humidified incubator at 37 °C with 5 % CO2. SiRNAs of human NEAT1 lentivirus and vector harboring green fluorescent protein (GFP) and the control lentivirus (GFP-lentivirus) were constructed by Genechem (Shanghai, China). The siRNAs sequences targeting NEAT1 were as follows: 5′-GUGAGAAGUUGCUUAGAAACUUUCC-3′ (siRNA-1) and 5′-GATCCCTAAGCTGTAGAACAT-3′ (siRNA-2). The lentiviruses for the overexpression and knockdown of miR-107 were provided by Genechem (Shanghai, China). The lentiviruses were diluted in 0.2 mL complete medium at 10^7^ transduction units (TU)/mL containing hexadimethrine bromide (Polybrene; 8 mg/mL) and were incubated with the cells for 1 h at 37 °C. Next, the cells were incubated with 0.3 mL fresh prepared Polybrene-Dulbecco’s modified Eagle’s medium for 24 h; the medium was then replaced with fresh Dulbecco’s modified Eagle’s medium and the cells were cultured for 48 h.

### Cell proliferation assay

The proliferation of Hep-2 cells was evaluated using cell count kit 8 (CCK-8) cell proliferation kit according to the manufacturer’s instructions. Briefly, the cells were seeded into 96-well plates at 5 × 10^3^ cells/well and cultured for 48 h. Then 10 μl CCK-8 solution were added to each well and incubated for 1 h. The absorbance (A) at 450 nm was measured using a microplate reader. Results were representative of three individual experiments in triplicate.

### Wound-healing migration assay

Cells were seeded in 6-well plates and grown to about 90 % confluence before being wounded using a 200-mL plastic tip across the monolayer cells. Debris was removed by washing three times with phosphate-buffered saline (PBS), and then cells were cultured with fresh DMEM containing 5 % FBS. Wound healing was observed at different time points within the scrape line, and representative scrape lines for each cell line were photographed. The migration of the cells was evaluated by measuring the width of the wounds (at × 400 magnification). Images were captured immediately after 24 h post-wounding. Each assay was performed in triplicate in at least two independent experiments.

### Transwell assay

Transwell filters (pore size, 8 μm; Falcon; BD Biosciences) were coated on the lower side with 8 μg/μL Matrigel and placed on a 24-well plate containing DMEM. Hep-2 cells (1 × 10^5^) were added to the upper compartment of a transwell chamber and allowed to migrate for 24 h at 37 °C. The cells were then harvested and suspended in DMEM with 1 % bovine serum albumin. After 24 h, Matrigel and cells remaining on the upper side of the membrane were wiped off with cotton swabs, and the cells that had migrated to the bottom surface of the membrane were fixed in 3.7 % paraformaldehyde in PBS. Once fixed, the cells were stained with crystal violet for 10 min at room temperature. Cell invasion was quantified by counting the number of cells in three inserts. Data were expressed as the average number of cells per insert.

### Cell cycle analysis

Cells were collected and fixed with cold ethanol at 4 °C for 1 h before being stored at −20 °C. Fixed cells were washed and resuspended in 1 mL PBS containing 50 μg/mL RNase A and 50 μg/mL ethidium bromide. After incubating for 20 min at 37 °C, cells were analyzed for DNA content by flow cytometry (FACS Calibur; Becton Dickinson Immunocytometry Systems, San Jose, CA, USA). For each sample, 20,000 events were acquired, and cell cycle distribution was determined using cell cycle analysis software (Modfit; LT for mac V 3.0).

### Apoptosis assay by flow cytometry

The cells were washed twice with cold 10 mM PBS and resuspended in 1 × binding buffer (BD Biosciences, San José, CA, USA) at a concentration of 1 × 10^6^ cells/ml. Cells were stained with Annexin V/ APC and propidium iodide (PI), using the Annexin V apoptosis detection kit (KeyGen Biotech, Nanjing, China). Hep-2 cells without any treatment were used as control, and the experiments were repeated at least three times.

### Animal experiments

Sixteen BALB/c mice (5 to 6 weeks old) were provided by Vital River Laboratories (Beijing, China) and kept in aseptic conditions with a constant humidity and temperature according to standard guidelines under a protocol approved by Harbin Medical University. All mice were injected subcutaneously in the dorsal scapula region with 100 μL suspension (1 × 10^6^) of Hep-2 cells. The size of the tumor was measured twice a week with calipers, and the volume of tumor was determined using the simplified formula of a rotational ellipsoid (length × width^2^ × 0.5). Once tumors reached approximately 0.5 to 0.6 cm^3^, mice received an injection into the tumor once a week for 3 weeks. Mice in the experimental group (*n* = 8) were treated with 100 μL NEAT1 siRNA lentivirus; mice in the control group (*n* = 8) received an injection of 100 μL GFP lentivirus. Tumors were harvested 1 week after the end of treatment.

### Transmission electron microscopy

Apoptotic morphological changes of cells were detected by transmission electron microscopy. Tumor tissues were fixed for 1 h with 2.5 % glutaraldehyde using 0.1 mol/L Na cacodylate buffer (pH 7.3), and then postfixed with 1 % osmium tetroxide for 1 h. Fixed samples were rinsed and dehydrated in a graded ethanol series. Ultrathin sections were cut, mounted on copper grids, and stained with uranyl acetate and lead citrate by standard methods. Stained grids were examined and photographed in a JEM 1200 EX transmission electron microscope (Tokyo, Japan).

### TUNEL assay

Apoptosis in vivo was detected using the terminal deoxytransferase-mediated dUTP nick end labeling (TUNEL) in situ apoptosis detection kit (Roche) according to the manufacturer’s instructions. After deparaffinization and dehydration and inactivation of peroxidase activity, 20 paraffin sections of each tumor specimen were incubated with 2 μg/mL proteinase K at 37 °C for 15 min. Then, the sections were treated with terminal deoxynucleotidyl transferase (TdT) and biotinylated dUTP, and observed under microscopy. Controls were treated in the same manner except that TdT was replaced with dH2O.

### Western blot analysis

Hep-2 cells were collected and CDK6 expression was detected as described previously using CDK6 antibody (1:400 dilution, Boster, Wuhan, China) [14]. Glyceraldehyde-3-phosphate dehydrogenase (GAPDH) was used as a loading control.

### Luciferase report assay

3′UTR of CDK6 gene harboring miR-107 binding site was inserted downstream of firefly luciferase (f-luc) in pGL3 plasmid and named as 3′UTR. 3′UTR of CDK6 gene with the deletion of miR-107 binding site was inserted downstream of f-luc in pGL3 plasmid and named as 3′UTR-NC. 3′UTR of CDK6 gene with three mismatch mutations in miR-107 seed complementary site was inserted downstream of f-luc in pGL3 plasmid and named as 3′UTR-MU. HEK293T cells were cultured in 96-well plates and co-transfected with 400 ng of either 3′UTR, 3′UTR-NC or 3′UTR-MU, 50 ng of pRL-TK (Promega, USA) and 50 nmol/L of miR-107 or scramble miRNA negative control (miR-NC). The pRL-TK Renilla luciferase plasmid was used as internal control. 48 h after transfection, firefly and Renilla luciferase activities were measured using the dual-luciferase reporter assay (Promega, USA). The results were expressed as relative luciferase activity (firefly luciferase/Renilla luciferase).

### Statistical analysis

All quantitative data were presented as the mean ± SD of at least three independent experiments. The differences between groups were analyzed by Student’s *t* test. *P* < 0.05 was considered as statistically significant.

## Results

### NEAT1 is overexpressed in LSCC

**Fig. 1:**
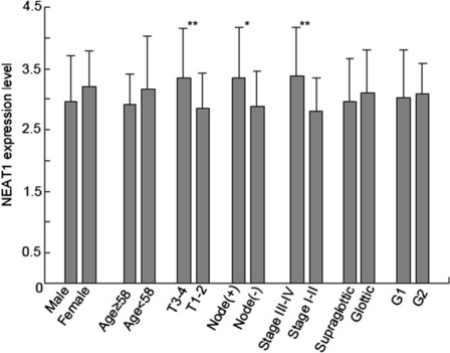
Expression of NEAT1 in LSCC tissues. Tumors with advanced clinical stages, with poor differentiation, with T3-4 grade or with lymph node metastasis expressed higher levels of NEAT1. *P < 0.05; **P < 0.01

qPCR analysis showed that NEAT1 levels were significantly higher in LSCC tumor tissues than in adjacent non-neoplastic tissues (3.041 ± 0.709 fold, *P* < 0.01). NEAT1 expression was significantly related with T grade, neck nodal metastasis, and clinical stage of LSCC (Fig. 1). Tumors with grade T3 to T4, lymph node metastasis, or advanced clinical stages expressed higher levels of NEAT1.

### NEAT1 knockdown inhibits the proliferation and invasion of LSCC cells

**Fig. 2:**
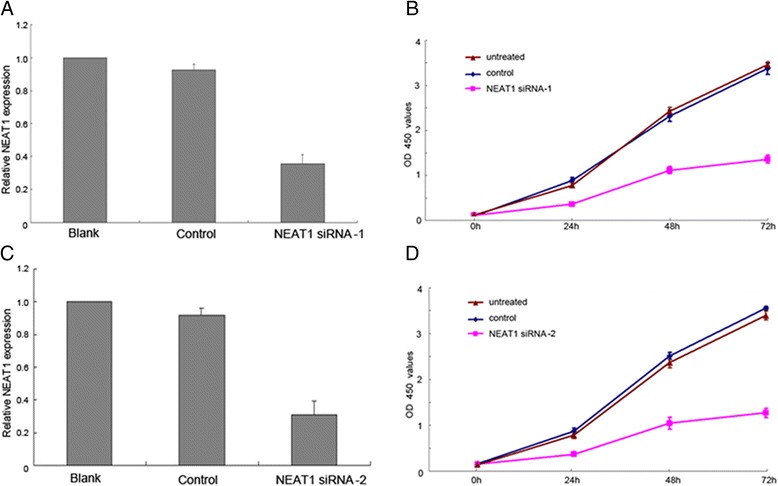
NEAT1 siRNA inhibited the proliferation of LSCC cells. **a** and **c** Expression of NEAT1 was significantly downregulated in Hep-2 cells transduced with two different NEAT1 siRNAs. **b** and **d** Cell proliferation was evaluated using CCK8 assay. The proliferation of Hep-2 cells transduced with two different NEAT1 siRNAs was decreased at different time point (24, 48, and 72 h, respectively) compared with the controls

**Fig. 3:**
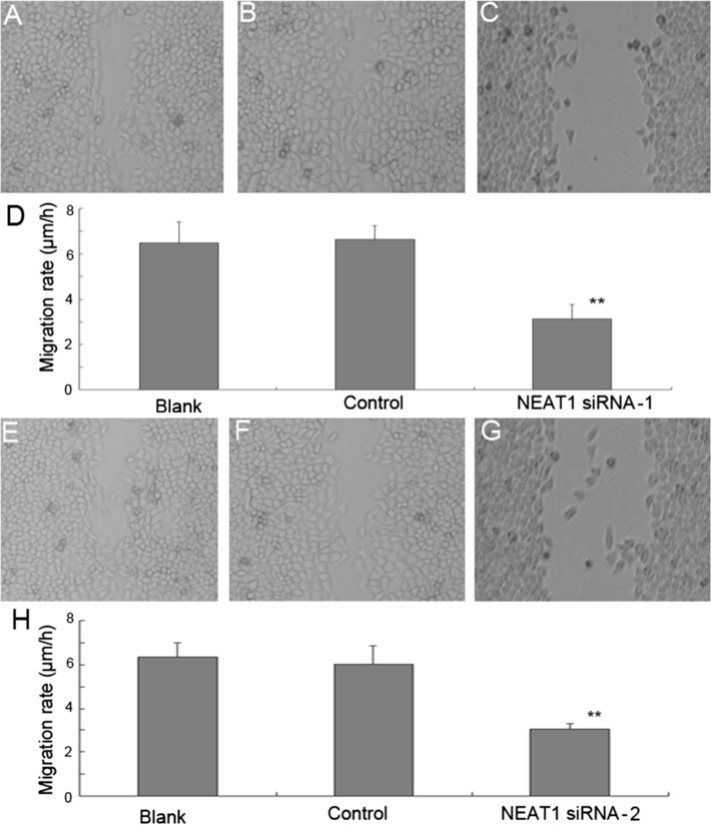
NEAT1 siRNA inhibited the migration of Hep-2 cells. Effect of two different NEAT1 siRNAs on cell migration was determined using scratch-wound healing migration assays, 24 h post-wounding. **a** and **e** Hep-2 cells without any treatment, **b** and **f** Hep-2 cells transduced with GFP vector control, **c** and **g** Hep-2 cells transfected with NEAT1 siRNA1 or siRNA2, **d** and **h** Cell migration was significantly repressed in Hep-2 cells transduced with NEAT1 siRNAs (**P < 0.01)

**Fig. 4:**
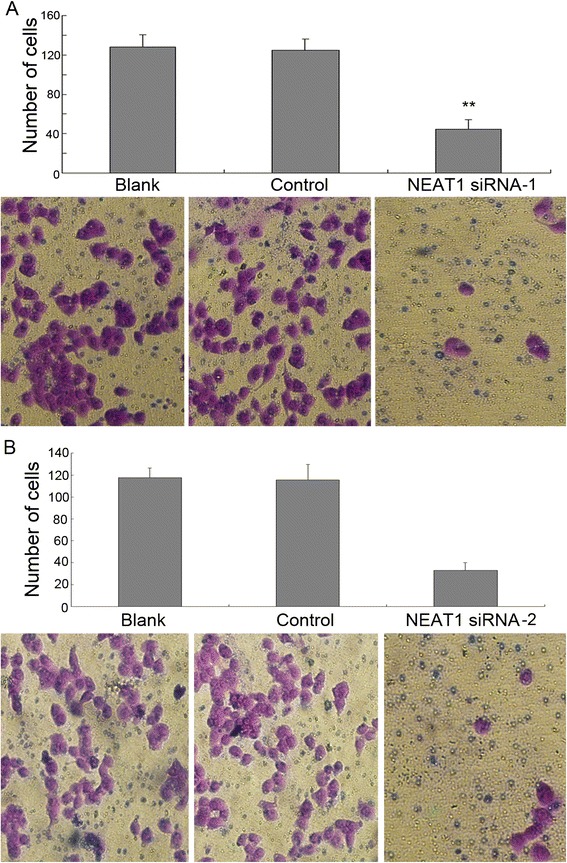
NEAT1 siRNA inhibited the invasion of Hep-2 cells. Transwell assay showed that after incubating for 24 h, the invaded cells that penetrated the lower surface of the membrane were significantly reduced compared to controls. **P < 0.01. **a** NEAT1 siRNA-1 group; **b** NEAT1 siRNA-2 group

Hep-2 cells transduced with NEAT1 siRNAs showed lower expression of NEAT1 compared with the control cells. CCK8 assay showed that NEAT1 knockdown inhibited Hep-2 cell proliferation at each time point (24, 48 and 72 h) (Fig. 2). By wound healing assay, we found that NEAT1 knockdown inhibited Hep-2 cell migration (Fig. 3). In addition, transwell migration and Matrigel invasion assay showed that NEAT1 knockdown inhibited Hep-2 cell migration and invasion (Fig. 4). Taken together, these results suggest that NEAT1 promotes the proliferation and invasion of LSCC cells.

### NEAT1 knockdown induces G1 phase arrest and apoptosis of Hep-2 cells

**Fig. 5:**
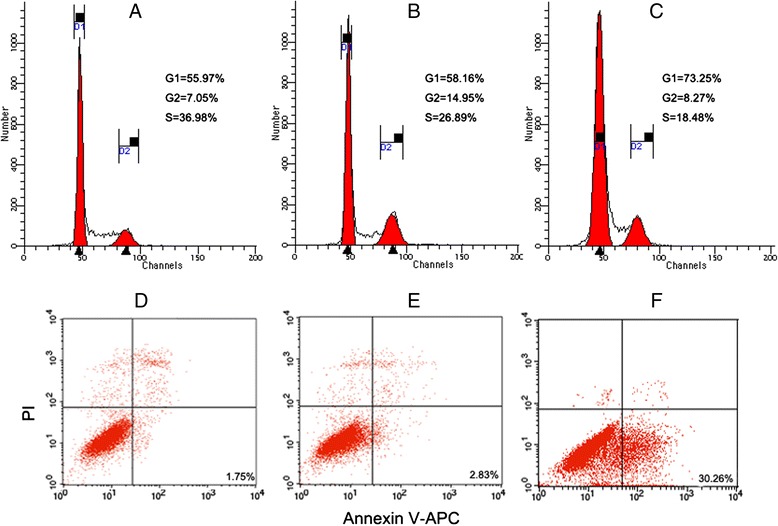
NEAT1 siRNA induced G1 phase arrest and apoptosis of Hep-2 cells. Flow cytometric analysis of the cell cycle of Hep-2 cells in each group: **a** blank control, **b** control lentivirus transduced Hep-2 cells, **c** NEAT1 siRNA lentivirus transduced Hep-2 cells. The dot plot of the X-axis (FL4) represented Annexin V fluorescence and the Y-axis (FL2) represented PI fluorescence. **d** Representative flow histograms of Hep-2 cells without any treatment. **e** Representative flow histograms of Hep-2 cells transduced with GFP-lentivirus control. **f** Representative flow histograms of Hep-2 cells transduced with NEAT1 siRNA lentivirus

Hep-2 cells transduced with GFP-lentivirus exhibited no significant changes in cell cycle progression at 72 h post-transduction compared to untransduced Hep-2 cells (*P* > 0.05). However, cells transduced with NEAT1 siRNA remained in the G1 phase compared to control cells (*P* < 0.05). Flow cytometric analysis showed that the percentage of apoptotic cells was significantly higher in NEAT1 siRNA transduced Hep-2 cells than in the cells transduced with GFP lentivirus (Fig. 5).

### NEAT1 knockdown inhibits the growth of LSCC xenografts

**Fig. 6:**
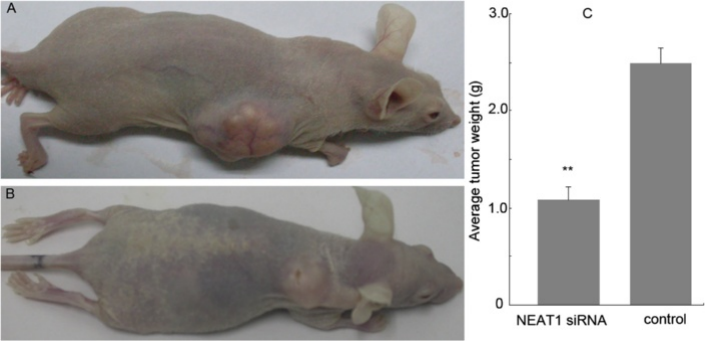
NEAT1 siRNA suppressed Hep-2 tumor growth in vivo. **a** Representative mouse injected with GFP control lentivirus. **b** Representative mouse injected with NEAT1 siRNA lentivirus. **c** Tumor weight in NEAT1 siRNA lentivirus-treated group was significantly less than in the control group. **P < 0.01. *n* = 8 mice per group

To provide in vivo evidence for the oncogenic role of NEAT1 in LSCC, we used a xenograft mice model. After the 16 mice were subcutaneously injected with Hep-2 cells, all of them developed detectable tumors. The growth of LSCC xenograft was significantly inhibited in mice treated with NEAT1 siRNA lentivirus, compared with mice treated with GFP lentivirus. The average tumor weight in NEAT1 siRNA-treated LSCC xenografts was significantly lower than that in the control group (1.085 ± 0.132 g versus 2.487 ± 0.160 g, *P* < 0.01) (Fig. 6).

### NEAT1 knockdown induces the apoptosis of LSCC cells in vivo

**Fig. 7:**
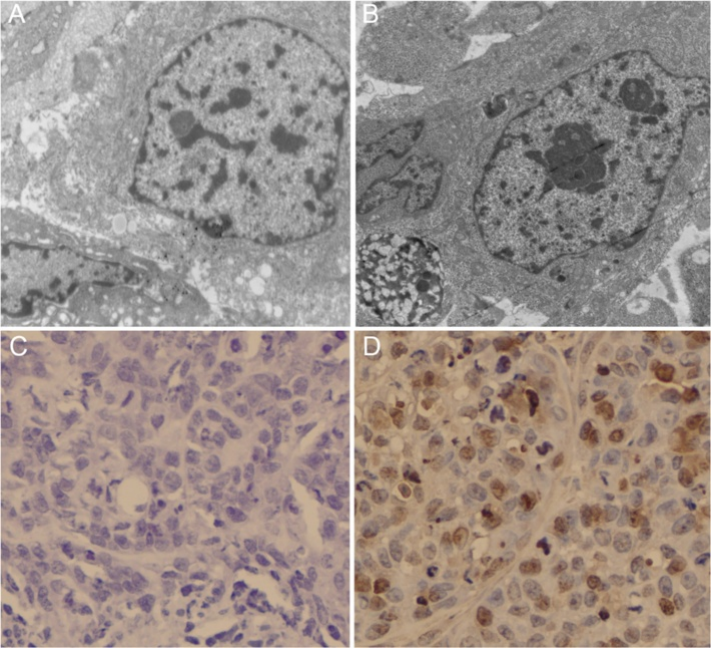
Apoptosis array in LSCC xenografts. **a** Cells in control Hep-2 xenografts had normal membrane, organelles, and nuclear morphology. **b** Apoptotic morphologies were found in NEAT1 siRNA treated Hep-2 xenografts. **c** TUNEL staining showed no obvious apoptotic cells in tumors of the control group. **d** Strong brown staining indicated many TUNEL positive apoptotic cells in NEAT1 siRNA treated Hep-2 xenografts

By transmission electron microscopy, we observed typical apoptotic morphology in the tumor cells in NEAT1 siRNA treated group, characterized by homogeneous condensation of chromatin to one side or the periphery of the nuclei. The inner matrix of some mitochondria showed increased electron density, typically observed in apoptotic cells. These ultrastructural changes were only observed in tumor cells of NEAT1 siRNA treated group but not in tumor cells of the control group, which showed intact membranes and intact morphology of the organelles. Furthermore, the apoptosis of tumor cells was detected using TUNEL assay, and the results showed strong TUNEL staining in NEAT1 siRNA-Lentivirus treated Hep-2 xenografts. In contrast, nearly no TUNEL-positive staining was detected in the control xenograft sections (Fig. 7).

### NEAT1 regulates CDK6 expression through modulating miR-107

**Fig. 8:**
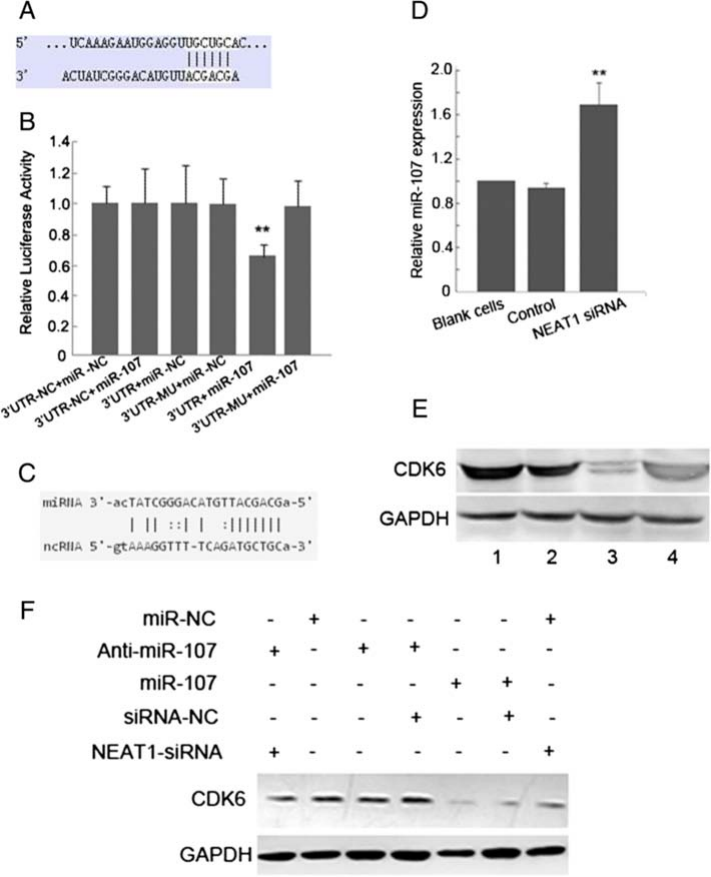
NEAT1 regulates CDK6 through modulating miR-107. **a** TargetScan database predicated CDK6 as a target of miR-107. **b** miR-107 decreased luciferase activity of 3′UTR but not that of 3′UTR-NC and 3′UTR-MU. miR-NC indicated scramble miRNA used as the negative control for miR-107. **P < 0.01. **c** Starbase database predicted the target site between CDK6 and miR-107. **d** Real-time PCR showed that miR-107 level significantly increased after NEAT1 knockdown in Hep-2 cells. **P < 0.01. **e** Western blot analysis showed that CDK6 protein level decreased after NEAT1 knockdown in Hep-2 cells. Lane 1 and 2: control Hep-2 cells; Lane 3 and 4: Hep-2 cells transduced with NEAT1 siRNA. GAPDH was loading control. **f** Western blot analysis showed that CDK6 protein level decreased after NEAT1 knockdown in Hep-2 cells, but was restored after the knockdown of miR-107. GAPDH was loading control

Based on two databases targetscan (www.targetscan.org) and starbase (starbase.sysu.edu.cn), we found that CDK6 is a potential target of miR-107 while NEAT1 may acts as a regulator of miR-107 (Fig. 8a and c). We speculated that there is a regulatory loop among CDK6, miR-107 and NEAT1 in LSCC cells. To confirm the direct interaction between miR-107 and its binding site within 3′UTR of CDK6, we created 3′UTR reporter plasmid and generated 3′UTR-NC and 3′UTR-MU plasmids as negative controls. Luciferase reporter assay showed that overexpression of miR-107 led to a marked decrease of luciferase activity of 3′UTR but could not decrease the luciferase activity of 3′UTR-NC and 3′UTR-MU (Fig. 8b). These results indicate that miR-107 directly modulate CDK6 expression by binding miR-107 seed complementary site located in 3′UTR of CDK6. Furthermore, RT-PCR and Western blot analysis showed that knockdown of NEAT1 in LSCC cells led to increased level of miR-107 and decreased level of CDK6 protein (Fig. 8d and e).

To further confirm NEAT1 can regulate CDK6 expression which is mediated by miR-107, we performed rescue experiments. In Hep-2 cells with high expression of NEAT1, overexpression of miR-107 led to significant reduction of CDK6 expression (Compared lane 2 to lane 5, Fig. 8f). In contrast, while NEAT1 knockdown decreased CDK6 expression (compared lane 7 to lane 2, Fig. 8f), downregulation of miR-107 (by lentivirus transduction of Anti-miR-107) could restore CDK6 expression inhibited by NEAT1 knockdown (compared lane 7 to lane 1, Fig. 8f). Taken together, these results suggest that NEAT1 regulates CDK6 expression through modulating miR-107.

## Discussion

lncRNAs are a class of noncoding RNA transcripts with no or little protein-coding capacity. Recent studies have suggested that more genomic sequences were transcribed into lncRNAs than protein-coding RNAs [15]. lncRNAs play important role in carcinogenesis [16]. Through regulating gene expression by a variety of mechanisms, such as transcription, post-transcriptional processing, chromatin modification and the regulation of protein function, lncRNAs are involved in multiple processes of tumorigenesis [17]. However, the function of lncRNAs in LSCC is not well characterized. Our previous studies showed that lncRNA HOTAIR played oncologic role in LSCC [18, 19]. In this study, by real-time PCR we found that lncRNA NEAT1 is overexpressed in LSCC. NEAT1 is transcribed from the familial tumor syndrome multiple endocrine neoplasia (MEN) type 1 locus on chromosome 11 [20]. Paraspeckles are a new class of subnuclear bodies and contain RNA-binding proteins in the interchromatin space of mammalian cells. Paraspeckle can regulate gene expression by retaining mRNAs for editing in the nucleus [21]. NEAT1 localizes to paraspeckles and serves as an architectural component of these nuclear bodies [22]. Recent studies showed that NEAT1 is upregulated and possesses oncogenic properties in various cancers such as lung, breast and prostate cancer [13, 20, 23]. In the present study, our data indicate that NEAT1 RNA is involved in LSCC aggressiveness. NEAT1 was upregulated in LSCC specimens compared with the control tissues. In particular, patients with grade T3 to T4, lymph node metastasis, poor differentiation, or advanced clinical stages expressed higher levels of NEAT1.

To further understand the biological function of NEAT1 in LSCC progression, both in vitro and in vivo assays were performed. Our data showed that siRNA mediated knockdown of NEAT1 led to significant inhibition of cell proliferation and invasion, while induced cell cycle arrest at G1 phase and apoptosis in LSCC cells. Furthermore, our results demonstrate that NEAT1 knockdown suppressed tumor growth in BALB/c mice xenografts and induced tumor cell apoptosis in vivo. These results indicate that high expression of NEAT1 promotes the progression of LSCC and NEAT1 plays oncogenic role in LSCC.

The molecular mechanism of NEAT1 is complex. Hirose et al. suggested that paraspeckles/NEAT1 attenuated the cell death pathway [24]. Choudhry et al. showed that NEAT1 expression correlated with poor survival of breast cancer and was induced by HIF-2 in hypoxia [20]. Chakravarty et al. identified NEAT1 as a potential target of ERα in prostate cancer [23]. Moreover, NEAT1 was reported to be regulated by miR-449a in lung cancer [25]. miR-107 belongs to the miR-103/107 family and functions as a tumor suppressor in multiple cancers including head and neck squamous cell carcinoma [26]. CDK6, a member of the CDK family, is a key regulator during the G1/S cell cycle transition. Cdk6 is highly expressed in head and neck squamous cell carcinoma and significantly correlates with tumor progression [27]. Targetscan predicted that CDK6 may be a target of miR-107 and Starbase database suggested the target sites between NEAT1 and miR-107. Thus, we speculated that NEAT1 can regulate CDK6 thorough miR-107 network in LSCC. Through luciferase reporter assay, we found that CDK6 is a direct target gene of miR-107. Furthermore, we showed that NEAT1 siRNA increased miR-107 level and decreased CDK6 protein level. These results are consistent with our predication and suggest that NEAT1 can regulate CDK6 through miR-107. These data provide new insights into noncoding RNA regulation network, suggesting that lncRNA not only regulate proteins but also modulate the target gene of miRNA indirectly through affecting miRNA expression.

## Conclusions

This is the first study to demonstrate oncogenic role of NEAT1 in LSCC. The molecular mechanism of NEAT1 function in LSCC is associated with the regulation of miR-107/CDK6 pathway. NEAT1 may serve as a marker for LSCC progression and a potential target for therapeutic intervention of LSCC.
